# Cordycepin induces apoptosis of human ovarian cancer cells by inhibiting CCL5-mediated Akt/NF-κB signaling pathway

**DOI:** 10.1038/s41420-018-0063-4

**Published:** 2018-05-23

**Authors:** Zhen Yang Cui, Soo Jung Park, Eunbi Jo, In-Hu Hwang, Kyung-Bok Lee, Sung-Woo Kim, Dae Joon Kim, Jong Chun Joo, Seok Hoon Hong, Min-Goo Lee, Ik-Soon Jang

**Affiliations:** 1 Department of Sasang Constitutional Medicine, College of Korean Medicine, Wonkwang University, Iksan, Jeonbuk 54538 Republic of Korea; 2 Department of Sasang Constitutional Medicine, College of Korean Medicine, Woosuk University, Wanju, Jeonbuk 55338 Republic of Korea; 3 Division of Bioconvergence Analysis, Korea Basic Science Institute, Daejeon, 305-333 Republic of Korea; 4 Neuroscience research institute, Korea University College of Medicine, Seoul, 02841 Republic of Korea; 5 Department of Biomedical Sciences, School of Medicine, University of Texas Rio Grande Valley, Edinburg, TX 78539 USA; 6 Department of Chemical and Biological Engineering, Illinois Institute of Technology, Chicago, IL 60616 USA

## Abstract

The chemokine, CCL5, is a key mediator for the recruitment of immune cells into tumors and tissues. Akt/NF-κB signaling is significantly activated by CCL5. However, the role of NF-κB inactivation in apoptosis induced by negative regulation of CCL5 remains unclear. Here, we analyzed the effect of cordycepin on NF-κB activity in SKOV-3 cells and found that cordycepin-mediated inhibition of NF-κB signaling induced apoptosis in SKOV-3 cells via the serial activation of caspases. In addition, immune-blotting analysis showed that CCL5 is highly expressed in SKOV-3 cells. In addition to activating caspases, we show that, cordycepin prevents TNF-α-induced increase in CCL5, Akt, NF-κB, and c-FLIP_L_ activation and that CCL5 siRNA could inhibit Akt/NF-κB signaling. Moreover, cordycepin negatively regulated the TNF-α-mediated IκB/NF-κB pathway and c-FLIP_L_ activation to promote JNK phosphorylation, resulting in caspase-3 activation and apoptosis. Also, we show that c-FLIP_L_ is rapidly lost in NF-κB activation-deficient. siRNA mediated c-FLIP inhibition increased JNK. SP600125, a selective JNK inhibitor, downregulated p-JNK expression in cordycepin-treated SKOV-3 cells, leading to suppression of cordycepin-induced apoptosis. Thus, these results indicate that cordycepin inhibits CCL5-mediated Akt/NF-κB signaling, which upregulates caspase-3 activation in SKOV-3 cells, supporting the potential of cordycepin as a therapeutic agent for ovarian cancer.

## Introduction

Cordycepin, 3′-Deoxyadenosine, is a known polyadenylation inhibitor with various pharmacological activities, such as anti-proliferative, anti-cancer, and anti-inflammatory effects^1–8^. Cordycepin is an active small molecule implicated in regulating various physiological functions by immune-activation and also presents various properties, including anti-viral, anti-infection, anti-inflammatory, anti-aging, anti-cancer properties, and enhances sexual performance^9–13^. Already, cordycepin has been shown to induce cancer cell death in a large spectrum of tumor cell lines, including breast^14^, colon^15^, and oral squamous cell carcinoma^8^. However, the effects of cordycepin in ovarian carcinoma cells are not clear until now. In some tissues, inflammatory conditions increase the risk of certain cancer. Cytokines and chemokines are involved in an intensive dialog enhancing angiogenesis, tumor metastasis, and the subversion of adaptive immunity, as well as changing responses to hormone therapy or to chemotherapy^16^. CCL5 belongs to the CC-chemokine family and plays a critical role in the migration and metastasis of human malignant tumor cells^17^. The activity of CCL5 is mediated by binding to CCR1, CCR3, and mainly CCR5^18, 19^. In the cancer microenvironment, cancer cell stimulates de novo secretion of CCL5 from cancer stem like cells (CSLCs), and CCL5 acts as a paracrine or autocrine signaling to promote tumor cell migration, invasion, and metastasis^20, 21^.

Akt /protein kinase B (PKB) is a crucial node in diverse signaling pathways essential in both normal cellular physiology, as well as various disease states. Akt signaling controls cell proliferation and anti-apoptosis, cell growth, glucose metabolism, cell migration, and metastasis. Akt is an integrative regulator of tumor survival and apoptosis, and it is also activated downstream of PI3K and is down-regulated by the cancer suppressor PTEN^22^. Akt functions through its ability to activate many key pro-oncogenic target genes that induce cell growth or antagonize apoptotic pathways.

Nuclear factor-κB (NF-κB) comprises a family of transcription factors that regulate the transcription of cytokines, antimicrobial effectors, and genes that control cellular differentiation, growth, and proliferation in cancer stem cells^23^. Inducible NF-κB activation relies upon phosphorylation-triggered proteasomal degradation of the inhibitor of NF-κB proteins (IκBs) that retain inactive NF-κB dimers in the cytosol in unstimulated cells^24^. Recent work suggests a role for NF-κB in the propagation of ovarian cancer cells, but the significance and mechanism of NF-κB in ovarian cancer remains poorly understood. The NF-κB pathway is overactivated in aggressive ovarian cancer^25^.

In this study, we used inflammatory mediator, *TNF-α*, which has been shown to participate in both the initiation and progression of cancer, and demonstrated that CCL5 is highly expressed in an ovarian cancer cell line under these conditions. We then investigated the functional mechanisms underlying the stimulation of the NF-κB signaling pathway by CCL5 in ovarian cancer cells. Herein, we show that cordycepin prevents constitutively Akt-mediated NF-κB transcription factor activation by downregulating CCL5 and that the consequent activation of JNK signaling pathway causes cancer cell death.

## Results

### Cordycepin inhibits the cell viability of ovarian cancer cells

**Fig. 1:**
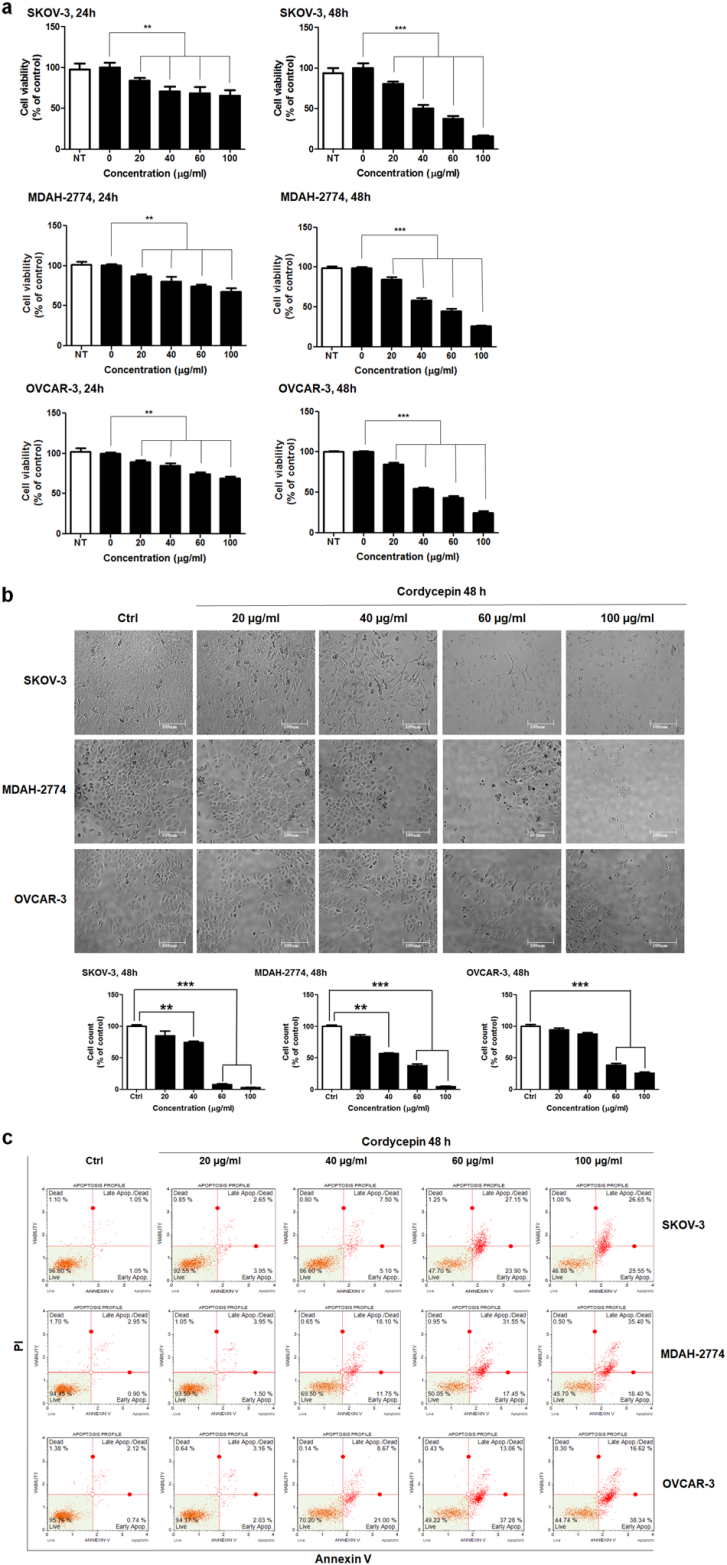
Cordycepin induces apoptosis in ovarian cancer cells. **a** Inhibition of the growth of SKOV-3, MDAH-2774, OVCAR-3 ovarian cancer cells by cordycepin. Ovarian cancer cells were exposed to 0, 20, 40, 60, and 100 μg/mL cordycepin for 24 and 48 h. **b** Microscopic images of SKOV-3, MDAH-2774, OVCAR-3 cells treated with cordycepin for 48 h. Magnification ×400. **c** Apoptosis analysis of ovarian cancer cells exposed to cordycepin. The statistics were shown the percentages of the cells represented by mainly early and late apoptosis which was apparent when the percentage of live cells markedly decreased. Data are presented as means ± standard deviations from triplicate experiments. Statistical significance was considered as **p < 0.01 and ***p < 0.001 vs. vehicle treatment

To investigate the effects of cordycepin on the proliferation of cancer cells. SKOV-3, MDAH-2774, and OVCAR-3 human ovarian cancer cells were treated directly with cordycepin at 0, 20, 40, 60, and 100 μg/mL for 24 or 48 h. As shown in Fig. 1a, cordycepin dose-dependently inhibited the cell viability of SKOV-3, MDAH-2774, OVCAR-3 during 24 h and 48 h incubation. At 40 μg/mL, 48 h treatment of cordycepin decreased approximately half of the SKOV-3, MDAH-2774, OVCAR-3 cell population (Fig. 1a). These results were consistent with cell viability of SKOV-3, MDAH-2774, OVCAR-3 cells treated with cordycepin. To observe the cell death of cordycepin-treated cancer cells, the morphologies of ovarian cancer cells were compared to those of untreated control cells by using light microscopy (Nikon TS-100, Nikon, Tokyo, Japan). The morphology of SKOV-3, MDAH-2774, and OVCAR-3 cells changed drastically after 60 μg/mL cordycepin treatment for 48 h. Multiple cells began to detach from the surface of the culture plate and appeared buoyant. Moreover, the cells appeared to be shrunken, resulting in reduced cell volume. These morphological changes preceded apoptosis. On the other hand, 40 μg/mL cordycepin induced less drastic changes at 48 h (Fig. 1b).

### Cordycepin induces apoptotic changes in ovarian cancer cells

The apoptotic effect of cordycepin on SKOV-3, MDAH-2774, OVCAR-3 cells were analyzed with Annexin V. For the evaluation of apoptosis, we used a Muse Annexin V and Dead Cell kit to measure the changes in cell apoptosis after 48 h. Ovarian cancer cell lines were treated for 48 h with 0, 20, 40, 60, and 100 μg/mL cordycepin. Total fractions of apoptosis (early and late apoptosis) were increased by cordycepin treatment in dose-dependent manner. The viable fractions of SKOV-3, MDAH-2774 and OVCAR-3 cells were reduced from 95, 94 and 97% in control group to 88%, 46% and 89% in cordycepin (100 μg/mL)-treated group, respectively (Fig. 1c).

### Effect of cordycepin on gene expression profiles

**Fig. 2:**
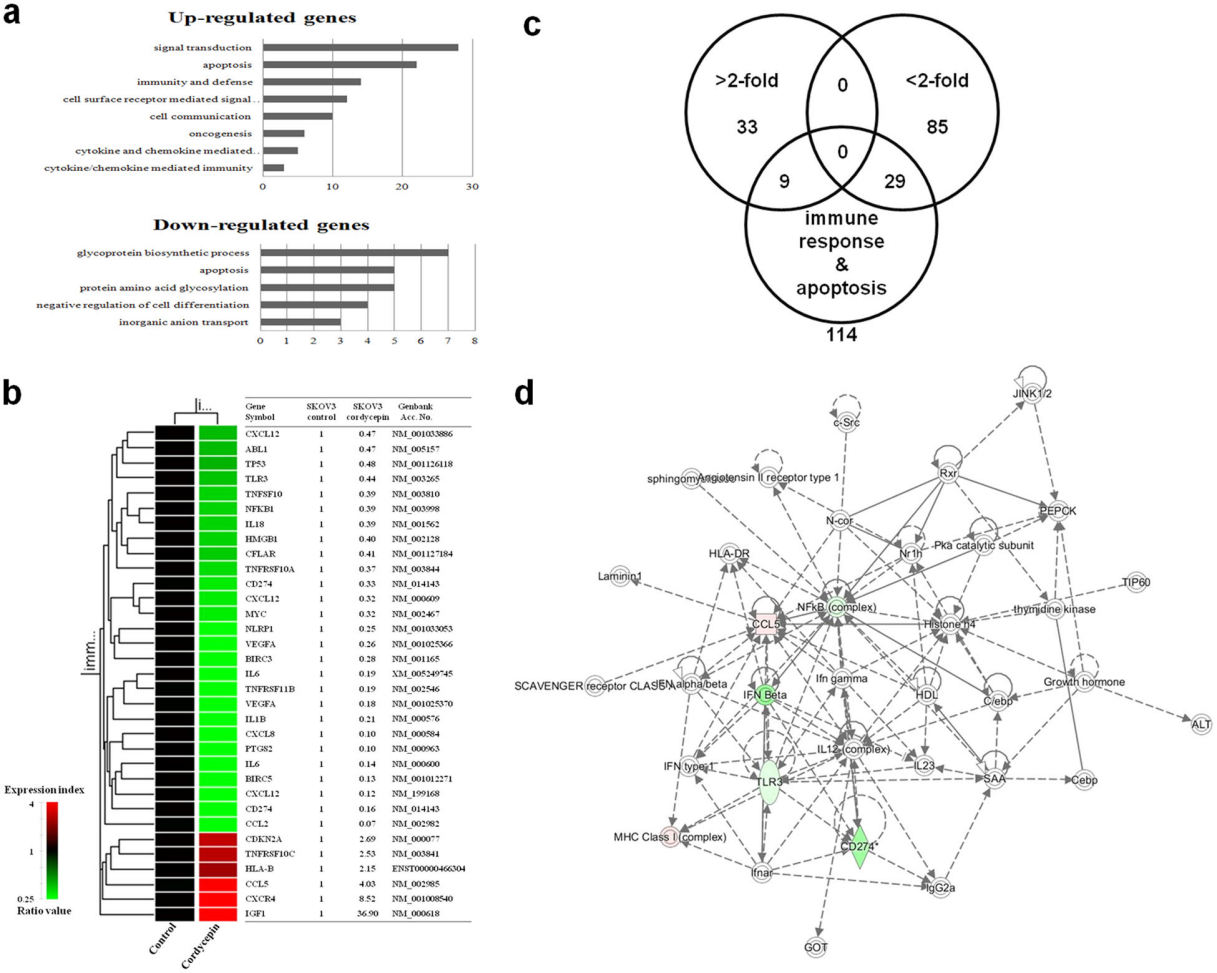
Gene expression analysis and signaling network of inflammation- and apoptosis-related genes. **a** Results of a gene ontology analysis by using microarray approaches in response to 100 μg/mL of cordycepin. Gene lists corresponding to two-fold upregulation and downregulation in cordycepin-treated SKOV-3 cells for 48 h were developed by using DAVID for Gene Ontology analysis (http://david.abcc.ncifcrf.gov/). **b** Immune response- and apoptosis-related genes and their hierarchical clustering in response to cordycepin. A dendrogram of hierarchical clustering revealed genes that were altered more than two-fold owing to apoptosis in response to cordycepin. **c** Gene lists (>2-fold, <2-fold, and apoptosis-related genes) are shown and are intersected individually by using Venn diagrams. **d** Signaling network of the immune response- and apoptosis-related genes in response to cordycepin. Nodes colored by using a Qiagen IPA were the genes in the apoptosis regulatory network in the cordycepin-treated SKOV-3 cells (red: upregulated genes, green: downregulated genes)

SKOV-3 cells were chosen for further study based on the results of cell viability test and FACs analysis. And SKOV-3 cells which have multiple characteristics of high grade serous, clear cell and endometrioid types were considered appropriate for a comprehensive view of anti-cancer effect of cordycepin across different ovarian cancer cell types. To assess the putative genes involved in cordycepin induced anti-tumor activity, we performed a gene expression microarray using SKOV-3 cancer cells treated with cordycepin. Among the 63,242 unique genes (using Agilent’s Human GE 8 × 60 K Microarray) tested, 30,858 genes were expressed in cells treated with 100 μg/mL of cordycepin. Among these 30,858 genes, 2561 and 1942 genes were upregulated and downregulated, respectively, by treatment with 100 μg/mL cordycepin compared to the untreated control at 48 h. Genes upregulated or downregulated by a 2-fold were further analyzed. Biologically relevant features were constructed by using the Database for Annotation, Visualization, and Integrated Discovery (DAVID) tools (http://david.abcc.ncifcrf.gov/). Gene lists corresponding to 2-fold up- and downregulation in the cordycepin-induced ovarian cancer cells were uploaded to DAVID for Gene Ontology analysis (Fig. 2a). To compare the results obtained upon cordycepin treatment with putative genes that belonged in apoptosis, we clarified candidate genes by the GeneCards database (http://www.genecards.org/) (Fig. 2b). The intersection obtained by hierarchical clustering is showed along with the gene lists in Fig. 2c. The signaling network of genes in response to apoptosis induced by cordycepin is presented in Fig. 2d.

### Cordycepin downregulates AKT/NF-κB signaling pathway and upregulates cleaved caspase-3

**Fig. 3:**
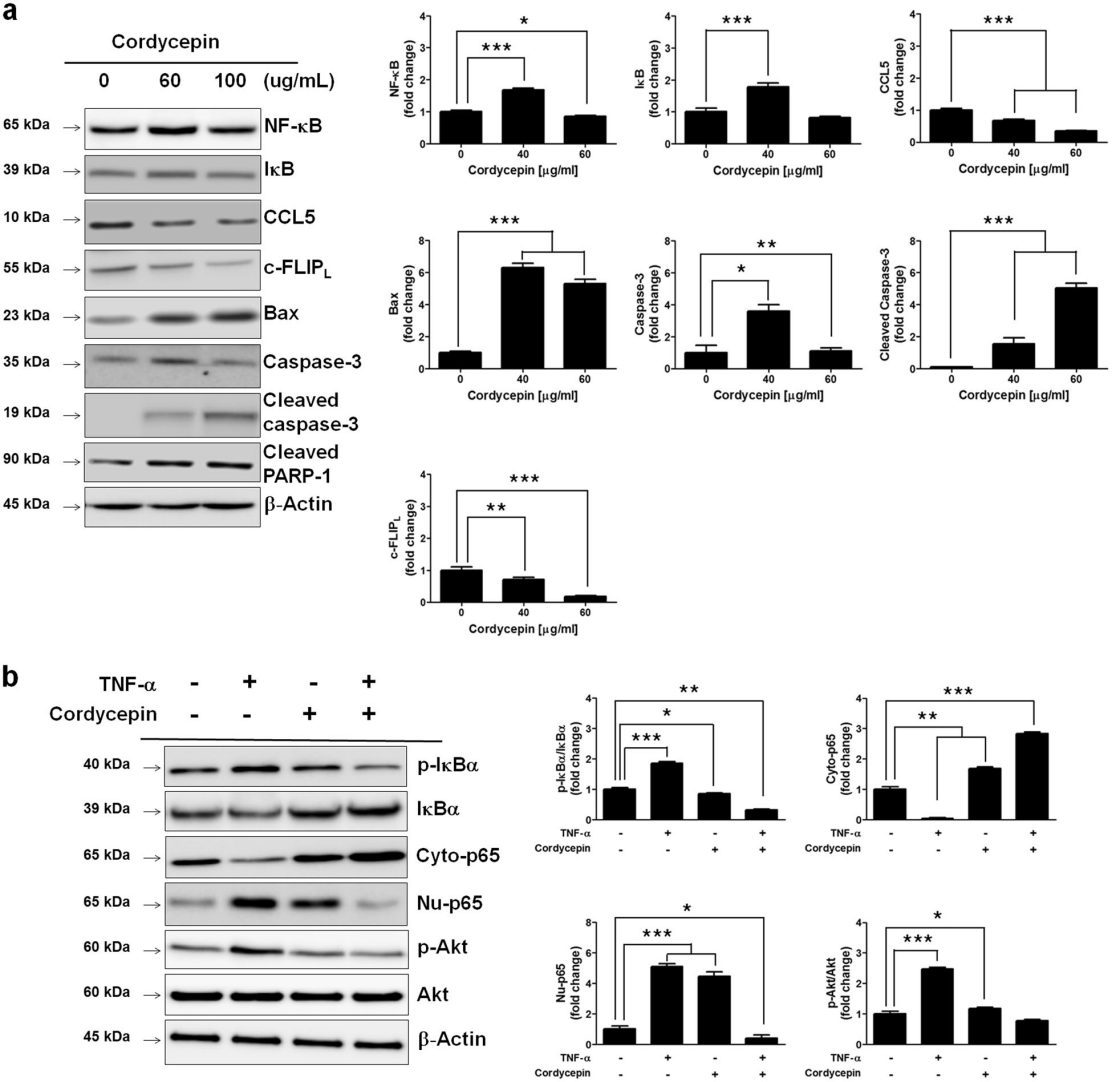
Inhibitory effect of cordycepin in TNF-α-mediated Akt/NF-κB signaling pathway. **a** Western blots showing the expression of NF-κB-p65, IκB, CCL5, c-FLIP_L_, caspase-3, and cleaved caspase-3 in response to 0, 60, and 100 μg/mL of cordycepin in SKOV-3 cells. **b** Effect of TNF-α on NF-kB was observed in SKOV-3 cells exposed to cordycepin. Western blot analysis was used to detect the expression of p-IκB, IκB, cytosolic NF-κB-p65, nuclear NF-κB-p65, p-Akt, and Akt in SKOV-3 cells cultured in the presence of 100 μg/mL of cordycepin. After 24 h, cells were challenged with 10 ng/mL of TNF-α for 24 h alone or in the presence of cordycepin. Data are expressed as means ± SD, * *p* < 0.05, ** *p* < 0.01, *** *p* < 0.001

Next, to investigate whether cordycepin suppresses the NF-κB signaling pathway, the expression of pro-inflammatory chemokine CCL5, IκB, NF-κB, c-FLIP_L_, Bax, cleaved PARP-1, and cleaved caspase-3 was determined in SKOV-3 cells exposed to cordycepin (Fig. 3a). Western blot analysis was used to detect the expression of NF-κB, c-FLIP_L_, Bax, cleaved PARP-1, and cleaved caspase-3 in SKOV-3 cells cultured in the presence of 60 and 100 μg/mL of cordycepin. As shown in Fig. 3a, increasing amounts of cordycepin resulted in a dose-dependent reduction in CCL5 expression, whereas NF-κB expression changed slightly at 60 μg/mL cordycepin, and decreased at 100 μg/mL cordycepin. Meanwhile, cordycepin treatment resulted in increased expression of cleaved caspase-3. As cordycepin induced apoptosis in ovarian cancer cells, it was appropriate to assess which downstream effectors mediated this process. NF-κB is a transcription factor that plays a important role in cytokine- and LPS-induced gene activation during inflammatory events^26^. This suggests that cordycepin blocked the inflammation-related signaling pathway such as CCL5, Akt/NF-κB, c-FLIP_L,_ and upregulated caspase-3 activation. The expression levels of inflammation-related proteins in SKOV-3 cells treated by TNF-α and cordycepin were determined by using western blotting assay. TNF-α is a cytokine that induces inflammation in ovarian cancer cells. Thus, we investigated the important role of cordycepin in TNF-α-treated SKOV-3 cells. Inflammation-related proteins such as Akt, IκBα, nuclear NF-κB, and c-FLIP_L_ were activated by TNF-α in SKOV-3 cells, while effectively downregulated by cordycepin in TNF-α treated SKOV-3 cells (Fig. 3b). The cordycepin (100 μg/mL) significantly decreased the expression of phosphorylated AKT and nuclear NF-κB increased by TNF-α treatment. These results indicate that cordycepin downregulated the TNF-α-mediated Akt**/**NF-κB/ c-FLIP_L_ signaling pathway in ovarian cancer cells.

### Cordycepin inhibits Akt/NF-κB signaling pathway through CCL5 and reduces migration

**Fig. 4:**
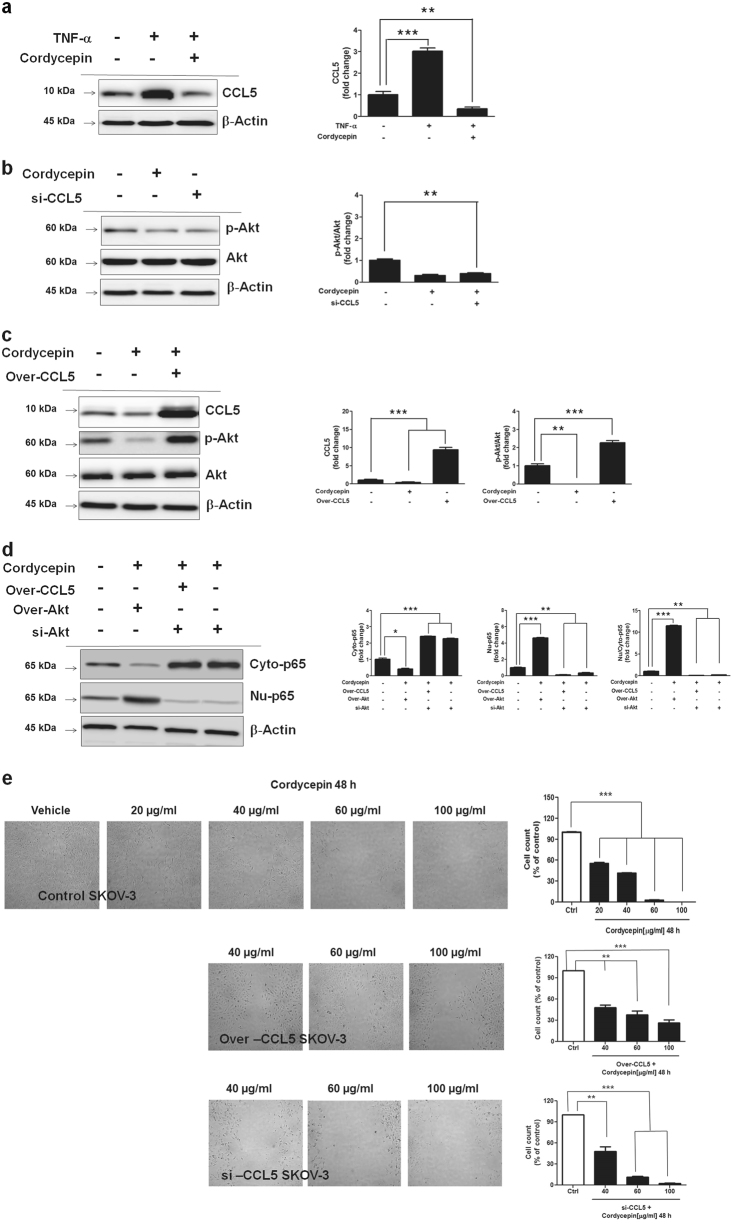
Attenuation of CCL5-induced Akt/NF-κB signaling by cordycepin. **a** Western blot analysis was used to detect the expression of CCL5 in SKOV-3 cells cultured in the presence of 100 μg/mL cordycepin. After 24 h, cells were challenged with 10 ng/mL of TNF-α for 24 h alone or in the presence of cordycepin. **b** SKOV-3 cells were treated for 24 h with cordycepin. After 24 h, cells were challenged with siRNA directed against CCL5 (si-CCL5) for 48 h alone or in the presence of cordycepin. **c** SKOV-3 cells were treated with cordycepin for 6 h, and the expression of CCL5, p-Akt and Akt was measured by western blotting assay. **d** SKOV-3 cells were treated with cordycepin for 6 h, then the medium was collected 24 h later and the secretion of cytosol and nuclear NF-κB was measured. SKOV-3 cells were incubated with siRNA directed against Akt (si-Akt) or negative control siRNA for 48 h, transfected with Akt and CCL-5-overexpression constructs for 48 h. Representative western blots were shown from 3 independent experiments. **e** Microscopic images demonstrating the results of the in vitro migration of SKOV-3 and CCL5-overexpressed and knock-downed SKOV-3 cells by using the simple scratch technique of migration assay. Data are expressed as means ± SD, *p < 0.05, **p < 0.01, ***p < 0.001

We detected CCL5 expression levels of SKOV-3 cells treated by TNF-α and cordycepin using western blotting assay (Fig. 4a). A significant (*p* *<* 0.05) increase of CCL5 release was observed in TNF-α-treated SKOV-3 cells. Meanwhile, the release of CCL5 was effectively downregulated by cordycepin treatment of TNF-α-treated SKOV-3 cells (Fig. 4a). Akt activation was also downregulated by cordycepin and a significant (*p* *<* 0.05) decrease of Akt activation was observed in SKOV-3 cells treated with CCL5 siRNA (Fig. 4b); whereas CCL5 overexpression increased Akt activation in cordycepin-treated SKOV-3 cells (Fig. 4c). These results indicated that cordycepin inhibits CCL5-mediated Akt signaling pathway in ovarian cancer cells. Next, we investigated the effect of Akt on NF-κB through cordycepin-regulated CCL5 in ovarian cancer cells. CCL5 effectively enhanced Akt phosphorylation in SKOV-3 cells. When Akt was silenced, cordycepin treatment of both SKOV-3 cells and SKOV-3 cells overexpressing CCL5 decreased nuclear translocation of NF-κB, whereas Akt overexpression increased nuclear translocation of NF-κB in cordycepin-treated SKOV-3 cells (Fig. 4d). To evaluate the potential biological relevance of the regulatory effect of cordycepin, we assessed the effect of CCL5 on the migration of tumor cells. Migration of SKOV-3 cells was measured by using a wound-healing assay. CCL5 overexpression increased cancer cell migration. The treatment of cordycepin (100 μg/mL) significantly suppressed the cell migration in both CCL5-overexpressed and control vector-transfected cancer cells in a dose-dependent manner at 48 h (Fig. 4e). These results indicate that cordycepin attenuates CCL5-mediated Akt/NF-κB phosphorylation to downregulate SKOV-3 cell migration.

### Cordycepin-mediated Akt/NF-κB/c-FLIP_L_ signaling inhibition activates JNK to induce caspase-3 activation

**Fig. 5:**
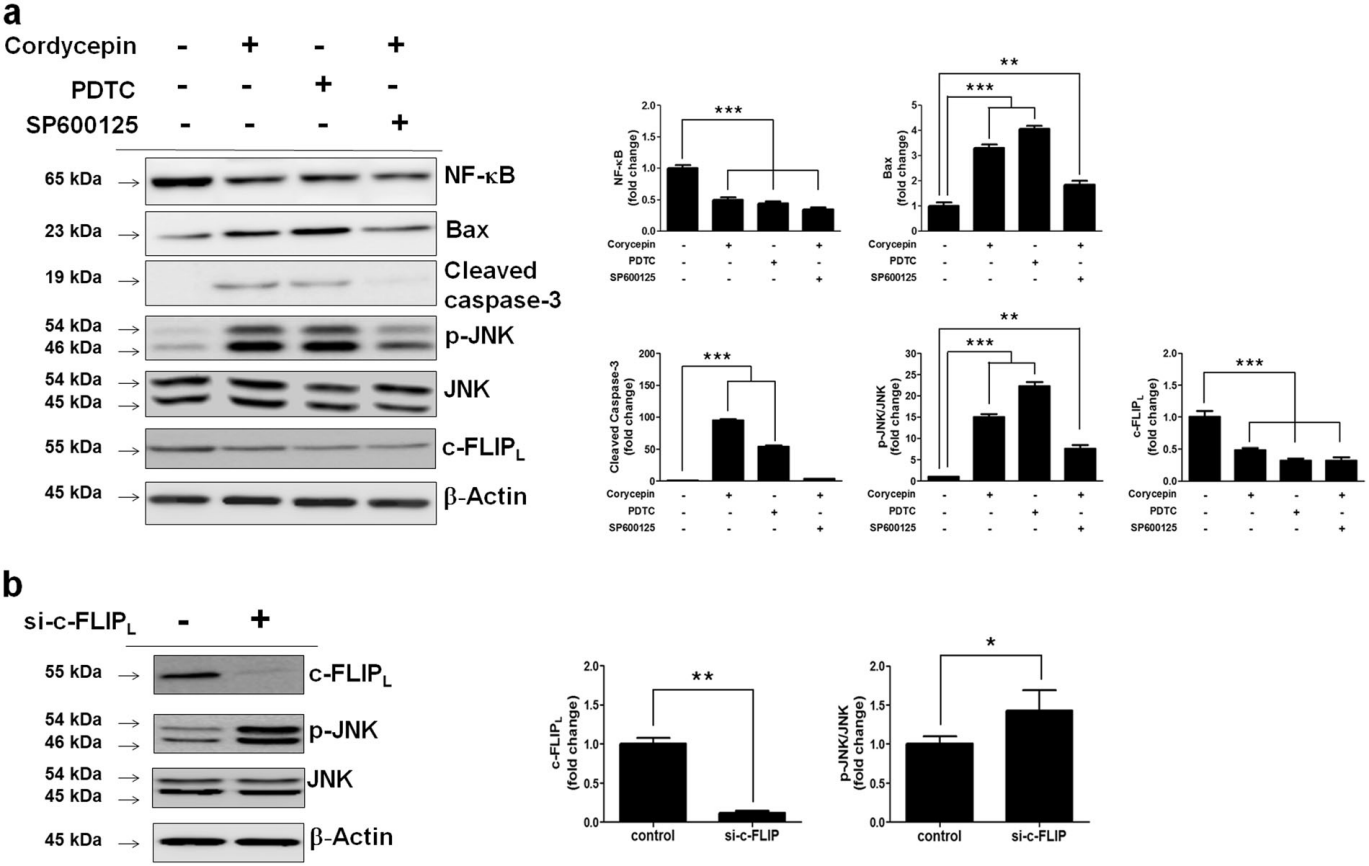
Promotion of JNK activation by cordycepin mediated c-FLIP_L_ inhibition. **a** SKOV-3 cells were treated with PDTC and SP600125 for 1 h, then cultured with cordycepin. The expression of NF-κB-p65, c-FLIP_L_, JNK, p-JNK and cleaved caspase-3 was analyzed by western blotting assay. **b** Cells were treated with si-c-FLIP_L_ for 1 day. The expression of c-FLIP_L_, JNK and p-JNK. Data are expressed as means ± SD, **p < 0.01, ***p < 0.001

To further investigate whether Akt/NF-κB is functionally linked to JNK signaling, we examined the effect of Akt/NF-κB on JNK. Cordycepin enhanced the expression of p-JNK. Next, we performed a loss-of-function experiment using NF-κB knockdown by cordycepin and the NF-κB inhibitor, PDTC. Both cordycepin and PDTC attenuated c-FLIP_L_ and enhanced p-JNK, and the selective JNK inhibitor, SP600125 (20 μM) reduced the phosphorylation of JNK and the expression of Bax. Immunoblots confirmed the reduction in p-JNK protein in SKOV-3 cells, whereas cordycepin enhanced the p-JNK protein-mediated increase in Bax and cleaved caspase-3 (Fig. 5a). Also, siRNA mediated c-FLIP inhibition increased JNK (Fig. 5b). These results indicate that JNK signaling promotes SKOV-3 cell apoptosis by enhancing caspase-3 activation. Taken together, these results indicate that cordycepin-mediated NF-κB inhibition upregulated p-JNK, leading to the upregulation of cleaved caspase-3. These results indicate that cordycepin suppresses the activation of Akt/NF-κB signaling pathway and c-FLIP_L_ activation, which results in p-JNK upregulation and the subsequent induction of caspase-3 activation.

## Discussion

Cordycepin has a variety of biological functions, including anti-tumor, antiviral, anti-oxidant, and anti-inflammatory activities^1, 13, 27, 28^. In the past few years, several reports indicated that cordycepin inhibits the expression of some inflammatory genes by suppressing NF-κB activation^29^. Meanwhile, other studies reported that cordycepin has anti-cancer and anti-metastatic effects, inhibiting the expression of some critical molecules involved in tumor growth and metastasis by blocking NF-κB activation^30, 31^.

In the present study, we showed that the CCL5-mediated Akt/NF-κB signaling pathway was involved in human ovarian cancer cells after cordycepin treatment. First, we found that cordycepin-mediated CCL5 inhibition downregulated p-Akt. Second, cordycepin-mediated Akt inactivation by inhibiting CCL5 reduced nuclear NF-κB preceded SKOV-3 ovarian cancer cell apoptosis. Third, cordycepin upregulated p-JNK through the reduction of nuclear NF-κB. Finally, treatment with the JNK inhibitor, SP600125, significantly decreased Bax and cleaved caspase-3. It has been reported that CCL5 acts through PI3K/Akt, which in turn activates IKKα/β and NF-κB, resulting in the activation of αvβ3 integrin and contributing to the migration of human lung cancer cells^32^. Therefore, to investigate whether cordycepin inhibits CCL5-mediated Akt/NF-κB signaling pathway, we detected CCL5 expression level in SKOV-3 cells treated by TNF-α and cordycepin using western blotting assay (Fig. 4a). CCL5 was increased in TNF-α treated SKOV-3 cells, while it was downregulated by cordycepin in TNF-α treated SKOV-3 cells (Fig. 4a). Akt activation was also downregulated by cordycepin and CCL5 siRNA in SKOV-3 cells (Fig. 4b); whereas CCL5 overexpression increased Akt activation in cordycepin-treated SKOV-3 cells (Fig. 4c). These results indicate that cordycepin could inhibit CCL5-mediated Akt signaling pathway in ovarian cancer cells.

A functional link exists between the Akt and NF-κB pathways and the Akt signaling pathway actively regulates NF-κB^33, 34^. Therefore, we investigated the effect of Akt on NF-κB through cordycepin-regulated CCL5 in ovarian cancer cells. Our results demonstrated that, when Akt was silenced, cordycepin decreased nuclear translocation of NF-κB, in SKOV-3 cells and SKOV-3 cells overexpressing CCL5, while Akt overexpression increased nuclear translocation of NF-κB in cordycepin-treated SKOV-3 cells (Fig. 4d), suggesting that cordycepin inhibits Akt/NF-κB signaling pathway through CCL5. FLIP is an important mediator of NF-kappaB-controlled antiapoptotic signals^35^.

We undertook a series of experiments to investigate whether c-FLIP is implicated in the antiapoptotic NF-κB response. Generally, activation of JNK is involved in the induction of apoptosis^36^, whereas c-FLIP exerts other cellular functions including increased cell proliferation and tumorigenesis^37, 38^.

Therefore, we evaluated the fundamental role of c-FLIP_L_ in the regulation of JNK signaling in SKOV-3 cells treated with cordycepin. c-FLIP_L_ was dramatically decreased, whereas JNK activation was increased in both PDTC- and cordycepin-treated SKOV-3 cells, whereas NF-κB activation was decreased and caspase-3 activation was increased (Fig. 5a). To further investigate the role of JNK in cordycepin-mediated apoptosis, we used the JNK inhibitor, SP600125, (20 μM) (Fig. 5a). SP600125 decreased the levels of Bax and cleaved caspase-3 in cordycepin-treated SKOV-3 cells. Also, siRNA mediated c-FLIP inhibition increased JNK (Fig. 5b). These results indicate that c-FLIP_L_ may therefore play a key role in the NF-κB-mediated control of death signals and that cordycepin augments Bax and caspase-3 activation through the activation of JNK by inhibiting NF-κB induced c-FLIP_L_ signaling.

We showed that the protein level of p-JNK was dramatically increased by negative regulation of CCL5-mediated Akt/NF-κB expression in cordycepin-treated SKOV-3 cells. We demonstrated that JNK is a critical mediator of cordycepin-induced SKOV-3 cell apoptosis. These findings provide novel insights into the molecular mechanisms of SKOV-3 cell apoptosis. Therefore, controlling CCL5 expression may provide new ways and strategies to enhance SKOV-3 cell apoptosis. Taken together, our results demonstrate that cordycepin mediates Bax’s apoptotic regulation of NF-κB by downregulating CCL5. Also, this systematic investigation study shows the precise molecular mechanism of NF-κB signaling pathway induced by cordycepin and reveals role and potential therapeutic use of cordycepin to inhibit migration in the treatment of ovarian cancer.

## Materials and methods

### Reagents and chemicals

Fetal bovine serum (FBS), 1% (w/v) penicillin-streptomycin and phosphate-buffered saline (PBS) were obtained from Thermo (Paisley, Scotland, UK). Dulbecco’s Modified Eagle’s Medium (DMEM) was purchased from Sigma-Aldrich (St Louis, MO, USA). Cordycepin (3′-Deoxyadenosine, from *Cordyceps militaris*), Pyrrolidine dithiocarbamate (PDTC), and SP600125 were purchased from Sigma-Aldrich. TNF-α was purchased from R & D system (R&D Systems, Minneapolis, MN, USA). A Muse Annexin & Dead Cell kit was from Millipore (Millipore, Billerica, MA, USA). Whole cell lysis buffer was purchased from Intron (Seoul, Korea), and transfection reagent Hilymax and CCK-8 were from Dojindo (Dojindo, Japan). Antibodies against CCL5, NF-κB, p-IκB, IκB, p65, p-Akt, Akt, caspase-3, and β-actin were purchased from Cell Signaling (Beverly, MA, USA). Antibodies against PARP-1 and Bax were from Santa Cruz Biotechnology (Dallas, TX, USA).

### Cell lines and cell viability assay

The human ovarian-carcinoma-cell line (SKOV-3, MDAH-2774, OVCAR-3) were obtained from the American Type Culture Collection (Rockville, MD, USA). Cells were grown in DMEM medium, supplemented with 10% (v/v) FBS and 1% (w/v) penicillin-streptomycin at 37 °C with 5% (v/v) CO_2_. Cells (5 × 10^3^ of cells per well) were placed in a 96-well plate. After a 24-h incubation, the cells were treated with cordycepin for 48 h. Cell viability assays were performed as previously described^39^. In brief, at the end of the treatment, 10 μL of cell-counting kit-8 solution were added to the cell solution and incubated for 1 h at 37 °C. Cell viability was determined by using a microplate reader (Sunrise, Tecan, Switzerland) to measure the absorbance at 450 nm. The assays were performed in triplicate. The appropriate dose was determined by evaluating the cytotoxicity of cordycepin for 48 h.

### Cell apoptosis assay

To detect the effect of cordycepin on apoptosis, we analyzed the Muse Annexin V & Dead Cell reagent (Millipore) following the user’s guide and the manufacturer’s instructions. Briefly, cells were treated with cordycepin for 48 h, harvested with trypsin-EDTA and washed twice in PBS. The cell suspension was centrifuged at 2000 rpm for 2 min and 1 × 10^6^ of cells were transferred in suspension with fresh medium containing serum to a new tube Staining protocol included warming the Muse Annexin V and Dead Cell Reagent to room temperature, addition of 100 μL of cells in suspension to each tube, addition of 100 μL of the Muse Annexin V and Dead Cell Reagent to each tube. Measurements were conducted by using an Muse Cell Analyzer (Millipore, Billerica, MA, USA). The statistics were shown the percentages of the cells represented by alive, apoptosis and dead population.

### Microarray analysis

For the microarray analysis of the cordycepin-treated SKOV-3 cancer cells, a human twin 44 K cDNA chip was used for the transcription profiling analysis. Total RNA was extracted from vehicle- or 100 μg/mL cordycepin-treated SKOV-3 cancer cells, and cDNA probes were prepared by using reverse transcription of 50 mg of RNA in the presence of aminoallyl dUTP, followed by coupling with Cy3 dye (vehicle) or Cy5 dye (cordycepin-treated). Genes were considered differentially expressed when, after a significance analysis of the microarray (SAM), the global M value, log_2_ (R/G), exceeded |1.0| (twofold) with a *P*-value < 0.05. A Student’s *t*-test was applied to assess the statistical significance of the differential expression of genes after cordycepin treatment. In order to analyze the biological significance of the changes, we categorized the array data into specific gene groups.

### Ontology-related network analysis

To study the biological functions of ontology-related regulated genes and proteins through their interaction network, we conducted a bioinformatic network analysis by using an ingenuity pathway analysis (IPA, http://www.ingenuity.com). The IPA identifies a gene interaction network based on a regularly-updated “Ingenuity Pathways Knowledge-base.” The updatable database was retrieved from the biological literature. Network generation was optimized from the inputted expression profile when possible and aimed at the production of highly connected networks.

### Fractionation and protein extraction

SKOV-3 cells were incubated with cordycepin for 2 days. The cells were collected with 2 mL of homogenization buffer A (25 mM Tris (pH 7.5), 2 mM EDTA, 0.5 mM EGTA, 1 mM DTT, protease inhibitor cocktail, 1 mM PMSF, and 0.02% Triton X-100) per culture dish, homogenized 15 times using a 15-mL Dounce homogenizer with pestle A, and centrifuged at 100,000 × *g* for 30 min. The supernatant cytosolic fraction was transferred into a new tube and 500 μL of homogenization buffer B (homogenization buffer A containing 1% Triton X-100) was added to the pellet. The pellet was resuspended by sonication, incubated for 30 min at 4 °C by rocking, and centrifuged at 100,000 × *g* for 30 min. The supernatant nuclear fraction was transferred into a fresh tube. The samples were prepared for protein analysis by western blotting.

### Western blot analysis

The expression of cordycepin-induced apoptosis-related signaling proteins was examined by using western blotting, as described previously^40^. In brief, 25 μg of the denatured protein was separated by using 12% polyacrylamide gel electrophoresis and transferred onto a nitrocellulose membrane. The nitrocellulose membrane was then stained with Ponceau S to position the proteins. The blotted membrane was blocked for 1 h with 5% (w/v) skimmed milk in TTBS (Tween-20 and Tris-buffered saline), followed by incubation with diluted primary antibodies, CCL5 (1:200), NF-κB(1:1000), p-IκB (1:500), IκB (1:500), p65 (1:1000), p-Akt (1:500), Akt (1:1000), caspase-3 (1:200), Bax (1:1000), PARP (1:1000), and β-actin (1:2000), at room temperature for 2 h or at 4 °C overnight. The membrane was washed three times for 5 min each time with 0.1% (v/v) Tween-20 in TBS before incubation with horseradish-peroxidase (HRP)-conjugated goat anti-mouse IgG or HRP-conjugated rabbit anti-goat IgG with a 1:2000 dilution in TBS containing 5% (w/v) skimmed milk at room temperature for 1 h. The membranes were rinsed three times with TTBS for 5 min each, and an enhanced chemiluminescence system (Thermo Scientific, San Jose, CA, USA) was used to visualize the bands on a ChemiDoc MP system (Bio-Rad, Hercules, CA, USA). Densitometric measurements of bands were performed by using Image J software (National Institutes of Health, Bethesda, MD, USA). The expression levels of proteins were quantitatively analyzed through comparison with actin used as an internal control.

### Biochemical analysis

CCL5 and AKT were overexpressed by using lentivirus (LV)-carrying RFP-conjugated full-length CCL5 or Akt (Lenti H1.4-ccl5/RFP, Lenti H1.4-Akt/RFP, Bioneer Corp., Daejeon, Korea). Small interfering RNAs (siRNAs) were purchased from ST Pharm (Seoul, Korea). The nucleotide sequence for Akt siRNAs was 5′-CGU UCU GCU GCG ACA AUG A-3′ and CCL5 siRNA (NM_001278736.1) was 5′-AAG GAA GUC AGC AUG CCU CUA-3′. SKOV-3 cells were seeded (2 × 10^5^ cells/6-well plates). After incubation, the cells were supplied with growth medium containing 10% FBS and were harvested 48 h later for further assays. siRNAs were transfected in SKOV-3 cells using lipofectamine RNAiMAX reagent (Invitrogen, Carlsbad, CA, USA) following the manufacturer’s instructions. Cells were then treated with 60 μg/mL of cordycepin for 48 h.

### Migration assay

The migration assay was conducted by using control RFP-vector-transfected and CCL5-RFP transfected cells. Cells were seeded into a 24-well plate. The cell monolayer was scraped with a pipette tip to create a wound. The cells were treated with 100 μg/mL of cordycepin for 48 h. The plates were imaged using the TissueFAXS system (TissueGnostics, Vienna, Austria). Cell migration was analyzed (quantification of the “healed” area and migrated cells) was performed with the HistoQuest software (TissueGnostics). Samples were analyzed with a Zeiss AxioImagerZ1 microscope system with a charge-coupled device camera and the TissueFAXS^TM^ automated acquisition system (TissueGnostics). The percentages of antibody-positive and stemness marker-positive tumors were determined and depicted as scattergrams. Images were digitalized and protein expression was quantified. Statistical flow analysis was performed with the HistoQuest^TM^ software (TissueGnostics).

### Statistical analyses

GraphPad Prism software (GraphPad, San Diego, CA, USA) was used for the statistical analyses. Student’s *t*-test was used to assess the statistical difference between the control and the MRGX-treated groups. *P* values < 0.05 were considered statistically significant.
